# Insights into environmental controls on microbial communities in a continental serpentinite aquifer using a microcosm-based approach

**DOI:** 10.3389/fmicb.2014.00604

**Published:** 2014-11-14

**Authors:** Melitza Crespo-Medina, Katrina I. Twing, Michael D. Y. Kubo, Tori M. Hoehler, Dawn Cardace, Tom McCollom, Matthew O. Schrenk

**Affiliations:** ^1^Department of Geological Sciences, Michigan State UniversityEast Lansing, MI, USA; ^2^Center for Environmental Education, Conservation and Research, Inter American University of Puerto RicoSan Juan, PR, USA; ^3^SETI InstituteMountain View, CA, USA; ^4^Exobiology Branch, Ames Research Center, NASAMoffett Field, CA, USA; ^5^Department of Geosciences, University of Rhode IslandKingston, RI, USA; ^6^Center for Astrobiology and Laboratory for Atmospheric and Space Physics, University of ColoradoBoulder, CO, USA

**Keywords:** serpentinization, subsurface, metabolism, nutrients, carbon

## Abstract

Geochemical reactions associated with serpentinization alter the composition of dissolved organic compounds in circulating fluids and potentially liberate mantle-derived carbon and reducing power to support subsurface microbial communities. Previous studies have identified Betaproteobacteria from the order Burkholderiales and bacteria from the order Clostridiales as key components of the serpentinite–hosted microbiome, however there is limited knowledge of their metabolic capabilities or growth characteristics. In an effort to better characterize microbial communities, their metabolism, and factors limiting their activities, microcosm experiments were designed with fluids collected from several monitoring wells at the Coast Range Ophiolite Microbial Observatory (CROMO) in northern California during expeditions in March and August 2013. The incubations were initiated with a hydrogen atmosphere and a variety of carbon sources (carbon dioxide, methane, acetate, and formate), with and without the addition of nutrients and electron acceptors. Growth was monitored by direct microscopic counts; DNA yield and community composition was assessed at the end of the 3 month incubation. For the most part, results indicate that bacterial growth was favored by the addition of acetate and methane, and that the addition of nutrients and electron acceptors had no significant effect on microbial growth, suggesting no nutrient- or oxidant-limitation. However, the addition of sulfur amendments led to different community compositions. The dominant organisms at the end of the incubations were closely related to *Dethiobacter* sp. and to the family Comamonadaceae, which are also prominent in culture-independent gene sequencing surveys. These experiments provide one of first insights into the biogeochemical dynamics of the serpentinite subsurface environment and will facilitate experiments to trace microbial activities in serpentinizing ecosystems.

## Introduction

Serpentinization is the aqueous alteration of low-silica ultramafic rocks characteristic of the lower oceanic crust and upper mantle. Water-rock reactions result in the transformation of olivine in the ultramafic rock to serpentine minerals, hydroxides, and magnetite, with concurrent increases in the alkalinity of the fluid. The mineral transformations associated with this process can produce significant quantities of H_2_, which in combination with CO and CO_2_, under highly reducing conditions, results in the formation of CH_4_ and other hydrocarbons through Fischer–Tropsch Type (FTT) synthesis (Abrajano et al., 1988; McCollom and Seewald, 2001). These reactions provide a source of energy and raw materials to support chemosynthetic microbial communities. However, serpentinizing environments are typically considered to be challenging for microbial life, due to their high pH, limited availability of electron acceptors, nutrients, and carbon sources.

Previous studies of the continental serpentinite subsurface environment focus on surface expression of the processes by sampling natural springs (Brazelton et al., 2012, 2013; Suzuki et al., 2013). To access directly the serpentinite subsurface environment, a microbial observatory was established in the Coast Range Ophiolite at the UC-Davis McLaughlin Natural Reserve in Lower Lake, CA (CROMO, Coast Range Ophiolite Microbial Observatory). CROMO consists of a series of wells drilled with the objective of monitoring groundwater biogeochemistry with limited microbial contamination (Cardace et al., 2013). Historical data from this ultramafic-hosted groundwater system was obtained from a series of environmental monitoring wells (henceforth referred to as pre-existing wells), drilled by the Homestake Mining Co., Inc., while conducting gold mining in the vicinity. The ultramafic-associated aquifer system is characterized by elevated pH, high Ca/Mg ratios, detectable dissolved H_2_, and elevated dissolved CH_4_ concentrations (Cardace et al., 2013).

A series of monitoring wells were drilled around two main boreholes (located ~1 km apart from each other), for a total of eight newly drilled wells (Cardace et al., 2013). The wells each have a low-flow membrane pump installed that allows direct access to the subsurface serpentinizing fluids. The periodic sampling of the fluid includes geochemical (organic and inorganic carbon, dissolved gases, nutrients) and microbiological (cell counts, DNA and RNA analyses, and culturing) characterization. The well fluids consist of generally low diversity microbial populations dominated by taxa within the class Betaproteobacteria and the order Clostridiales, consistent with previous studies of serpentinite ecosystems (Brazelton et al., 2012, 2013; Suzuki et al., 2013; Tiago and Veríssimo, 2013). However, most of these organisms have eluded cultivation attempts, and it remains unclear what metabolisms and controlling factors shape these communities.

Metagenomic analyses of microbial communities in serpentinite springs of the Tablelands Ophiolite in Newfoundland, Canada found evidence of betaproteobacterial populations with the potential to fix CO_2_ and assimilate CO, coupled with hydrogen oxidation (Brazelton et al., 2012). Culture-independent studies by Tiago and colleagues of deep continental wells associated with serpentinization in Portugal found sequences related to *cbbL*, a gene associated with autotrophic carbon fixation, and to Euryarchaeota related to the putative methane-oxidizing ANME-1 group (Tiago and Veríssimo, 2013). Recently, several novel betaproteobacterial strains, within the family Comamonadaceae, closely related to environmental sequences commonly obtained from a number of serpentinite ecosystems, were isolated from ultrabasic springs at “The Cedars” in California, USA (Suzuki et al., 2014). These isolates, referred to as “*Serpentinomonas*” are able to use a variety of carbon sources including small organic acids and solid calcium carbonate. Additionally, *Dethiobacter alkaliphilus* of the order Clostridiales, related to sequences commonly obtained from continental serpentinites, was found to assimilate small organic acids, such as acetate, while gaining energy from the anaerobic respiration of hydrogen (Sorokin et al., 2008). However, significant gaps remain in connecting these observations to the activities of microbial communities in serpentinization-associated ecosystems.

To understand better the factors limiting microbial growth in CROMO serpentinizing fluids, a comprehensive sampling campaign was conducted in March 2013 to produce co-registered sets of data related to microbial diversity and nutrient geochemistry. In parallel, microcosm experiments were set up with samples collected during two separate sampling campaigns in March and August 2013. Baseline geochemical data including pH, oxidation-reduction potential (ORP), dissolved oxygen (DO), and temperature were collected, in addition to gas geochemistry and nutrient analysis. During these experiments factors such as nutrients, electron acceptors, and carbon sources were investigated. Together, these data provide insight to controlling factors upon microbial populations in serpentinite fluids, and their relationship to microbial activities.

## Materials and methods

### Sample collection

Fluid and gas samples for wells CSW 1.1, N08B, and N08C were collected using positive displacement Teflon bladder pumps from Geotech Environmental Equipment. Well fluids were pumped through an YSI 3059 flow cell, after air bubbles were flushed out of the system. Standard geochemical measurements (temperature, specific conductance, pH, DO, and ORP) were then collected using a YSI 556 multiprobe when the DO measurement stabilized at a minimum value, generally after ~5 min, or roughly 3–4 l of pumping. The outlet of the flow cell was fitted with silicone tubing, to which was fixed a 1 mL syringe tip, which allowed for easier collection of fluid samples by connection to a three-way luer-lock stopcock mated with a 60 mL syringe. Again, care was taken during sample collection into the syringe to ensure that there were no remaining gas bubbles in the syringe that could lead to apparent loss or gain of dissolved gases.

Fluid samples for well CSWold were collected using a previously installed high-flow conventional deep well submersible pump. The standard geochemical measurements (temperature, specific conductance, pH, DO, and ORP) were collected using an YSI 556 multiprobe by inserting the probe directly into the stream of water emanating from the 2-inch wellhead. Records were taken when the DO measurement stabilized at a minimum value, generally after ~1 min, or roughly 12–22 L of pumping. Samples were collected by inserting 60 mL syringes directly into the wellhead and slowly drawing fluid into them to avoid vacuum degassing of the sample.

Two 1 L pyrex bottles were filled free of headspace with fluid from each well and capped with a 43 mm butyl rubber stopper. During the March 2013 sampling campaign, samples from the pre-existing wells N08B and CSWold were collected and during the August 2013 campaign, in addition to N08B and CSWold, one of the newly drilled wells, CSW1.1 was collected. Fluid samples were kept at 4°C until further processing. Microcosms enrichments were set up no later than a week after sample collection (see Materials and Methods above).

### Geochemical characterization

Dissolved gas phase samples (CH_4_, CO, H_2_) were collected by first extracting the dissolved gases from the fluid sample. Fifty mL of well fluids were pumped into a bubble-free 60 mL luer tip syringe, through a three-way stopcock fixed to the luer fitting, then 10 mL of instrument grade nitrogen (99.9995%) was injected through the three-way stopcock and taken up into the 60 mL syringe. The gas/fluid mixture was then shaken vigorously for 60 s, which allowed the dissolved gases to partition from the aqueous phase into the preferred gas phase of the nitrogen. After 60 s, shaking stopped and the resulting gas was injected into an evacuated 60 mL Calibond-5 gas sampling bag for analysis. All gas phase samples were collected in triplicate, with daily blanks collected from the nitrogen cylinder and stored in the Calibond-5 gas sampling bags.

Aqueous phase samples for dissolved inorganic carbon (DIC) and organic acids, were collected by directly pumping into a bubble-free 30 mL luer tip syringe, through a three-way stopcock fixed to the luer fitting. For DIC, 30 mL of well fluids were pushed through a 0.2 μM syringe filter (Whatman™ Puradisc 25 mm PES sterile packed, GE Healthcare Life Sciences, Pittsburgh, PA) into a pre-calibrated and nitrogen flushed 125 mL glass serum bottle (Wheaton Industries, Inc., Millville, NJ) fitted with a 20 mm thick blue butyl stopper (Chemglass Life Sciences, Vineland, NJ) with a vent needle to allow for excess nitrogen headspace to escape. Organic acid samples were collected by pushing 15 mL of well fluid through a 0.2 μM syringe filter (as above) into acid washed and ashed 20 mL I-Chem™ vials with PTFE lined caps. All aqueous samples were collected in duplicate.

Dissolved gas samples were analyzed for CH_4_ via a SRI 8610C GC-FID. Briefly, 100 μL of gas was removed from the gas sampling bag and injected directly onto a 1/8″ × 1 meter silica gel column, allowing for separation of CH_4_, CO, and CO_2_, then analyzed by FID. The same sample bags were analyzed for H_2_ and CO via a Trace Analytical RGA3 Reduced Gas Analyzer. Briefly, 25 μL of gas was removed from the sampling bag and injected directly onto a 1/8″ × 6 foot Carbosphere packed column (Alltech Associates, Inc., Deerfield, IL) followed by a 1/8″ × 2.5 foot Molsieve 5A packed column before being analyzed by the reduction gas analyzer detector.

Upon return to the lab, all DIC samples were immediately acidified with 3 mL of 85% phosphoric acid, which liberates the DIC as CO_2_ and preserves the samples from microbial action. The resulting CO_2_ headspace was analyzed via a SRI 8610 GC-FID, with the detector set to methanize CO_2_ to CH_4_ via H_2_ addition in the presence of a nickel catalyst at 380°C.

Organic acid samples were analyzed by HPLC, adapted from a method using 2-nitrophenylhydrazide derivatives (Albert and Martens, 1997). Our method differs from that of the Albert and Martens by incorporation of an automated sampler, for higher throughput sampling. All sample vials were analyzed with duplicate injections.

The filtrate from the 0.2 μm Sterivex filter cartridges (EMD Millipore, Billerica, MA) used for molecular sampling (see Materials and Methods above) was collected into a 30 mL Nalgene bottle for dissolved organic carbon (DOC), nitrogen and phosphorous species: total dissolved nitrogen (TDN), ammonium (NH^+^_4_), nitrite (NO^−^_2_), nitrite + nitrate (NO^−^_2_ + NO^−^_3_), total dissolved phosphorous (TDP), and phosphate (PO^−3^_4_). Samples were kept cold in the field and frozen at −20°C immediately upon arrival to the field station. Samples were sent for analysis at JBL Analytical Services (University of Georgia, Athens, GA).

### Cell counts

Fluids were preserved in 3.7% formaldehyde for cell abundance enumeration and stored at 4°C until processing. The preserved fluids were filtered through a 0.2 μm filter, and cells stained with 1 μg/ml of 4′,6-diamidino-2-phenylindole (DAPI) and were counted by epifluorescence microscopy using appropriate filter sets according to previously published protocols (Hobbie et al., 1977; Schrenk et al., 2003). Number of generations (n) were calculated following according to the following equation:

n = 3.3×log(b/B)

Where b equals end point cell counts and B equals the number of cells at the beginning of the incubation. Statistical significance of the end point cell counts between treatments were calculated by doing a *t*-test using the GraphPad Software (GraphPad Software, Inc., La Jolla, CA, USA).

### DNA extraction

One to four liters of samples were filtered through a 0.2 μm Sterivex filter cartridges (EMD Millipore, Billerica, MA). Filters were flash frozen in liquid nitrogen and stored at −80°C until processing. DNA extraction followed previously described protocols (Huber et al., 2002; Sogin et al., 2006). DNA extracts were purified with the DNA Clean and Concentrator™-5 kit (Zymo Research, Irvine, CA) according to the manufacturer's instructions. DNA extracts were quantified using a Qubit® 2.0 Fluorometer (Life Technologies, Grand Island, NY, USA).

### 16S rRNA gene enumeration (qPCR)

Copies of the 16S rRNA gene were estimated by quantitative polymerase chain reaction (qPCR) with the SsoAdvanced SybrGreen Assay by BioRad (Hercules, CA) using bacterial-specific primers targeting the V6V4 region of the 16S rRNA gene (Sogin et al., 2006).

### 16S rRNA gene tag sequencing and data analysis

DNA samples, from March 2013, were submitted to the Marine Biological Laboratory's (MBL) Josephine Bay Paul Center for amplicon sequencing of the V4V5 region of the 16S rRNA gene on an Illumina MiSeq instrument (Morrison et al., 2013; Huse et al., 2014b). These samples included N08C, N08B, CSWold, and CSW1.1. N08C was included for comparison purposes, since the geochemical characteristics of this sample are more moderate than any of the other wells tested.

Sequences were subjected to quality control and preprocessing in accordance with MBL's standard methods on their VAMPS server (Huse et al., 2014a). Sequence reads were aligned to the SILVA SSURef alignment v102 and taxonomic classification was assigned using Mothur v1.32.1 (Pruesse et al., 2007; Schloss and Westcott, 2011). Sequences were clustered into operational taxonomic units (OTUs) at the 97% similarity threshold using the cluster.split command and the average-neighbor clustering algorithm in mothur (Schloss et al., 2009). Alpha-diversity (within sample) was assessed by rarefaction analysis, while beta-diversity (between sample) of the microbial communities was assessed by calculation of the Morisita–Horn index and displayed in a dissimilarity dendrogram produced with the tree.shared command in mothur (Schloss et al., 2009). MatGAT (Campanella et al., 2003) was used to determine the percent sequence identify of the most abundant OTUs compared with 16S rRNA sequences, trimmed to the V4V5 region of the gene, from isolates within this study and from previous studies of the serpentinite subsurface (Tiago and Veríssimo, 2013; Suzuki et al., 2014). Taq sequence data generated by the MBL and associated with this study can be found in the VAMPS database (http://vamps.mbl.edu) under the project code DCO_BRZ.

### Microcosm experiment set up

In the lab, 50 mL of fluid were transferred to 160 mL serum bottles inside an anaerobic chamber under a N_2_:H_2_ (99:1) atmosphere to avoid excessive exposure to oxygen. The bottles were capped with butyl rubber stoppers and crimp sealed. Three bottles per treatment were prepared, in addition to a filtered control to monitor possible contamination. A 15 mL subsample was fixed for initial (*T* = 0) cell counts and the remaining fluid was filtered through a 0.2 μm Sterivex filter for DNA extraction.

For the March 2013 microcosm experiments, 20 mM sodium thiosulfate and 0.5 mM sodium sulfide was added as electron acceptor to all bottles. Treatments with nutrients included 1× N/P solution (which included 3 mM postassium phosphate dibasic and 0.4 mM ammonium chloride). Two mM sodium acetate was added to acetate containing treatments. Headspace for these incubations was H_2_:Ar mix (50:50, v/v) for the acetate treatment or H_2_:CO_2_ mix (80:20, v/v) for the autotrophic treatments, at 1.4 atm overpressure (see **Table 2**).

For the August 2013 microcosm experiment, no N/P solution or additional electron acceptors were added. Treatments included the addition of 2 mM CH_4_, 2 mM sodium acetate, or 2 mM sodium formate with an H_2_:Armix (50:50, v/v) headspace, or the addition of H_2_:CO_2_ mix (80:20, v/v) to the headspace at 1.4 atm overpressure (see **Table 2**).

The microcosms were incubated for 12 weeks and were sampled every 4 weeks to monitor growth via cell counts (2 mL). At week 12 the incubation were stopped, an end point cell count sample was collected, pH was recorded, sample was preserved for microbial isolations and the remaining fluids from the triplicate experiments were combined into one 0.2 μm Sterivex filter for DNA extraction following Materials and Methods described above. Filters were stored at −80°C until further analysis, which included 16S rRNA gene quantification by qPCR and endpoint sequencing.

### Endpoint DNA sequencing

The 16S rRNA gene was amplified in triplicate using primers Bac27F (5′-AGAGTTTGGATCMTGGCTCAG-3′) and Univ1492R (5′-CGGTTACCTTGTTACGACTT-3′), and GoTaq® DNA Polymerase (Promega Corporation, Madison, WI, USA) for the end point microcosm DNA extracts. PCR protocol consisted in an initial denaturation at 94°C for 5 min, followed by 30 cycles of 30 s at 94°C, 45 s at 54°C, and 2 min at 72°C with a final extension of 10 min at 72°C. Triplicate reactions were combined and purified using the QIAquick PCR Purification Kit (Qiagen, Santa Clarita, CA, USA).

Purified PCR products were send for sequencing at East Carolina University Genomics Core Facility. Sequencing results were visualized using FinchTV v1.5.0 (Geopiza, Inc., Seattle, WA), assembled, when possible with CAP3 sequence assembly program and screened for their closest relatives using the BLAST application of the NCBI database.

## Results

### Geochemical characterization of the fluids

Fluids from CROMO wells are reducing, compared to the more moderate well, with low ORP (ranging from −65.3 to −292.8 mV), pH ranging between 9.8 and 12.2 and fluid temperature fluctuating between 14.7 and 18.2°C (Table 1). N08C well is a more moderate well, with highly variable ORP value (−127.6 in March and 217.3 in August 2013 samples), pH between 7.4 and 7.6 and temperatures of 14.6–15°C (Table 1).

**Table 1:** **Geochemical characterization of CROMO fluids**.

	**CSWold**	**CSWold**	**CSW1.1^a^**	**CSW1.1**	**N08B**	**N08B**	**N08C^a^**	**N08C^a^**
Month	March	August	March	August	March	August	March	August
pH	9.8	9.8	12.2	12.4	10.9	11.0	7.4	7.6
Temperature (°C)	17.7	18.2	14.9	16.2	14.7	16.0	14.6	15.0
ORP (mV)	−220.4	−277.5	−292.8	−254.9	−126.5	−65.3	−127.6	217.3
DO (mg/L)	8.80	0.02	0.50	0.20	0.38	0.31	3.12	0.17
Conductivity (mS/cm^3^)	8.2	11.2	4.5	4.5	2.9	3.1	0.8	1.1
H_2_ (μM)	<0.003	<0.003	0.0128	1.4822	0.1501	<0.003	<0.003	0.3813
CO (μM)	<0.003	0.011	0.104	0.012	0.028	<0.003	0.029	0.017
CH_4_ (μM)	1680	1983	455	613	338	98	<0.003	<0.003
DIC (μM)	63	42	210	194	22	21	1831	1144
DOC (μM)	27	93	1163	989	67	104	408	211
TDN (μM)	1934	1560	1192	1070	221	231	127	52
NH^+^_4_ (μM)	2067	2022	1130	1093	232	222	1.0	7
NO^−^_2_ (μM)	0.06	0.05	0.08	bdl^b^	0.04	0.09	0.08	1.28
NO^−^_3_ + NO^−^_2_(μM)	0.23	0.16	0.22	0.61	0.20	0.20	98.80	41.98
TDP (μM)	0.33	1.44	0.77	1.32	0.04	0.03	1.60	2.01
PO^3-^_4_ (μM)	0.53	0.51	0.89	0.47	0.11	0.12	1.55	1.94
Lactate (μM)	<0.88	<0.04	<0.88	0.6	<0.88	<0.04	<0.88	0.2
Acetate (μM)	<0.92	<0.41	57.8	49.4	<0.92	0.7	<0.92	<0.41
Formate (μM)	<0.65	<2.25	19.8	5.1	<0.65	<2.25	<0.65	<2.25
Propionate (μM)	0.4	0.4	4.2	3.4	0.4	0.3	0.4	0.3
Butyrate (μM)	<2.16	<3.21	63.8	47.3	<2.16	<3.21	<2.16	<3.21
Valerate (μM)	0.2	<0.14	2.1	<0.14	<0.06	<0.14	<0.06	<0.14

*Average geochemical properties of the well water samples collected during March and August 2013. NO8C well is used as a control well for comparison purposes*.

a *No microcosm experiment was done from these samples*.

b *bdl, below detection limit*.

The fluid from the CSWold well had the highest methane concentration, ranging from 1680 to 1983. Five μM, while the methane concentration in the N08C well was below detection (0.003 μM). In contrast, N08C well had the highest DIC concentration (1144–1830 μM) while the concentration in the other wells ranged from 21.3 to 209.7 μM. CSW1.1 well is the most extreme well, with pH between 12.2 and 12.4 and ORP values between −292.8 and −254.9. The fluids from this well had the highest DOC concentration (989–1163 μM) and highest organic acid concentrations (compared to the others). The well fluids were also enriched in phosphorous and nitrogen, ammonium being the dominant nitrogen species, which is in contrast with other terrestrial serpentinizing sites, where nitrogen and phosphorous species are below detection (Tiago et al., 2004; Morrill et al., 2013).

### Culture-independent sequencing

16S rRNA gene tag sequence data, obtained from DNA extracted from the CROMO fluids collected in March 2013, indicate that the fluids from the high pH wells had much lower diversity, compared to the most moderate well, N08C (Supplementary Figure 1). The most extreme sample (CSW1.1) had the lowest diversity of all, as shown by the rarefaction curve (~100 OTUs per 250,000 reads, Supplementary Figure 1A). Replicate samples from each well are more similar to each other than samples from other wells, as indicated by their beta-diversity represented by a dissimilarity dendrogram (Supplementary Figure 1B).

**Figure 1:**
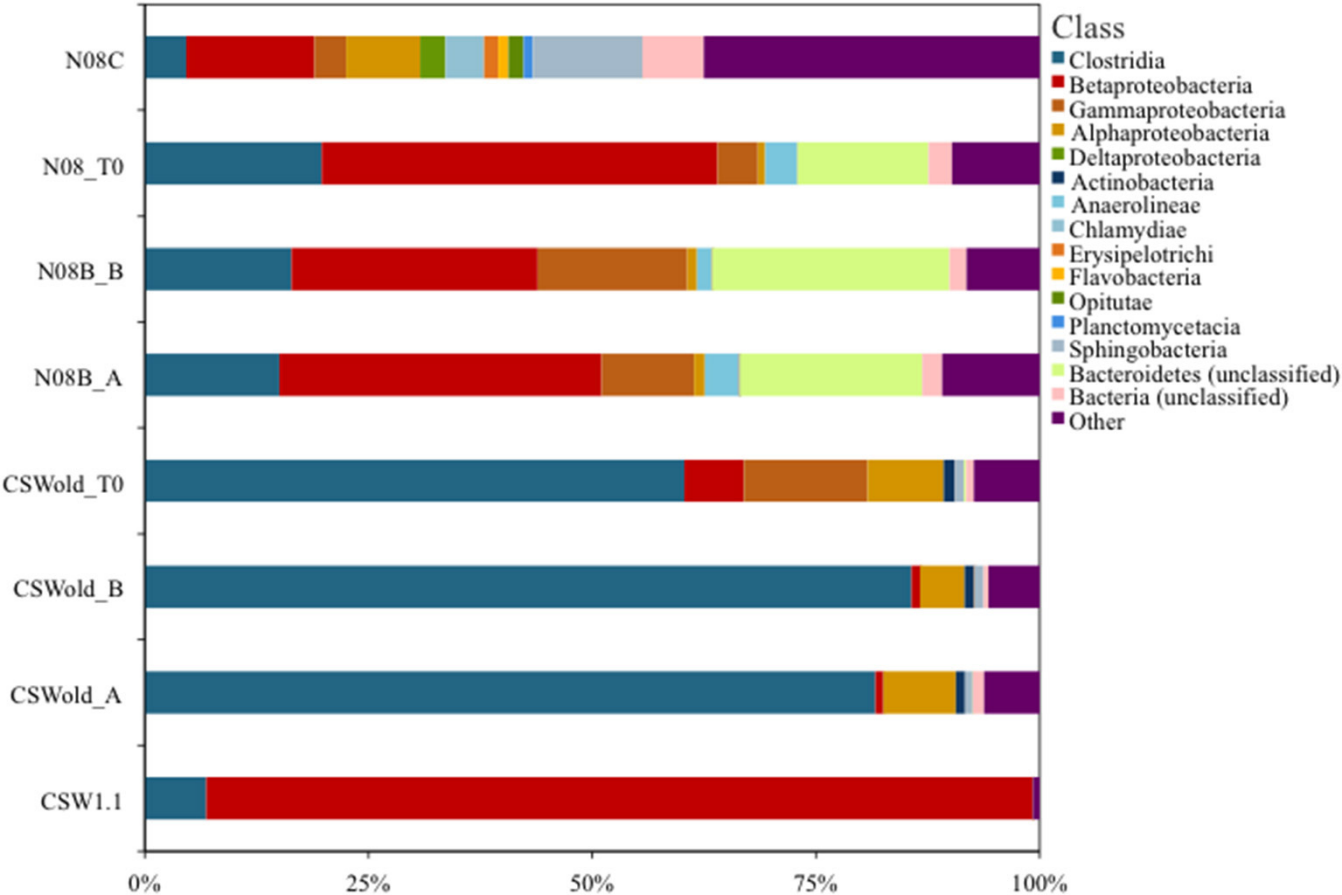
**Community composition at class level**. Proportion of bacterial classes present in fluids collected at CROMO in March 2013. Field replicate samples are indicated by A or B after the sample name. OTUs were made at 97% similarity level and assigned taxonomy from the SILVA (v102) database using Mothur v1.32.1 (Pruesse et al., 2007; Schloss and Westcott, 2011). The CSWold and N08B T0 samples show a slight shift in community composition compared to their field replicate counterparts.

A single OTU related to Betaproteobacteria dominated the 16S rRNA tag sequence libraries from CSW1.1 (>85% of the reads) and from N08B (~30–50% of the reads) (Figure 1; Supplementary Figure 2). The single betaproteobacterial OTU (OTU001) from CSW1.1 and N08B_T0 (Supplementary Figure 2) shows 100% sequence identity to clone sequences from Portugal (Tiago and Veríssimo, 2013) and isolate sequences from “The Cedars” (Suzuki et al., 2014; **Table 5**). OTUs related to the Betaproteobacteria correspond to less than 10% of the reads from the CSWold libraries, which were dominated mostly (60–80%) by OTUs related to the order Clostridiales (Figure 1). The most abundant Clostridia OTU, OTU002, was identified as *Dethiobacter* and comprised 85% of the Clostridia in CSW_T0 (Supplementary Figure 2). OTU002 shares 97% sequence identity with *D. alkaliphilus* and 99% sequence similarity with a 16S rRNA clone from a serpentinite site in Portugal (Tiago and Veríssimo, 2013; **Table 4**).

### Microcosm results

#### March microcosms

Microcosm experiments were set up with well water samples according to Table 2. For the microcosms established with the March samples, we tested the effect of nutrient and organic carbon (acetate) additions to the fluids. In general, for N08B microcosms, the addition of nutrients (N/P solution) had no significant effect on growth, while acetate stimulated growth over CO_2_, as can be observed from the difference in end point cell counts (*p-value = 0.0025)* (Table 2), as well as from the DNA yield per mL of sample and 16S rRNA gene quantification (Table 2). The addition of nutrients to the CO_2_ microcosm produced a slight (but not significant) increase in end point cell counts, DNA yield, and 16S rRNA gene copies per mL of sample. Addition of nutrients to the acetate treatments decreased the end point cell counts (Table 2), while DNA yield decreased from 10 to 2.9 ng per mL of sample and the 16S rRNA gene copies decreased an order of magnitude (Table 2).

**Table 2:** **Microcosm incubations set up and result for both March and August experiments**.

	**Sample**	**Carbon source**	**Nutrients**	**Electron acceptor**	**pH**	**Average cell counts^a^ (cells/mL)**	**n^b^**	**Volume filtered (mL)**	**Total DNA (ng)**	**DNA yield (ng/ml)**	**Bacterial 16S rRNA (copies/ml)**
March	N08B	^–^	^–^	^–^	10.8	6.57 × 10^5^		700	6380	9.1	5.80 × 10^6^
		CO_2_	No addition	Na_2_S_2_O_3_/Na_2_S	7	8.8 × 10^4^ (3.8 × 10^4^)	−2.9	700	6380	9.1	5.80 × 10^6^
		CO_2_	K_2_HPO_4_/NH_4_Cl	Na_2_S_2_O_3_/Na_2_S	7	1.8 × 10^5^ (5.3 × 10^4^)	−1.9	125	398.3	3.2	5.03 × 10^6^
		Acetate	No addition	Na_2_S_2_O_3_/Na_2_S	9	2.5 × 10^6^ (6.1 × 10^5^)	1.9	127	453.3	3.6	7.63 × 10^6^
		Acetate	K_2_HPO_4_/NH_4_Cl	Na_2_S_2_O_3_/Na_2_S	9.5	4.2 × 10^5^ (3.4 × 10^5^)	−0.6	125	1245	10.0	5.46 × 10^7^
	CSWold	^–^	^–^	^–^	9.8	2.71 × 10^4^		700	996.8	1.4	1.09 × 10^6^
		CO_2_	No addition	Na_2_S_2_O_3_/Na_2_S	7	4.4 × 10^4^ (7.2 × 10^3^)	0.7	125	23.3	0.2	1.12 × 10^5^
		CO_2_	K_2_HPO_4_/NH_4_Cl	Na_2_S_2_O_3_/Na_2_S	7	1.1 × 10^5^ (3.1 × 10^4^)	2.0	127	128	1.0	2.76 × 10^6^
		Acetate	No addition	Na_2_S_2_O_3_/Na_2_S	10	8.4 × 10^4^ (1.4 × 10^4^)	1.6	125	453.4	3.6	4.04 × 10^7^
		Acetate	K_2_HPO_4_/NH_4_Cl	Na_2_S_2_O_3_/Na_2_S	9.5	3.0 × 10^5^ (7.5 × 10^4^)	3.4	126	358.3	2.8	1.07 × 10^6^
August	N08B	^–^	^–^	^–^	10.9	7.48 × 10^5^		920	981	1.1	3.81 × 10^6^
		CO_2_	No addition	No addition	7	2.1 × 10^5^ (7.5 × 10^4^)	−1.8	123	361	2.9	7.24 × 10^6^
		CH_4_	No addition	No addition	10	1.9 × 10^6^ (1.3 × 10^6^)	−1.3	123	596	4.8	1.46 × 10^7^
		Acetate	No addition	No addition	10	1.2 × 10^6^ (7.7 × 10^5^)	0.7	124	694	5.6	1.15 × 10^7^
		Formate	No addition	No addition	10	8.3 × 10^5^ (6.7 × 10^5^)	0.1	125	544	4.4	8.84 × 10^6^
	CSWold	^–^	^–^	^–^	9.8	2.00 × 10^4^		630	602.5	1.0	3.29 × 10^6^
		CO_2_	No addition	No addition	7	1.1 × 10^5^ (4.8 × 10^4^)	2.4	123	25.4	0.2	2.87 × 10^4^
		CH_4_	No addition	No addition	9.5	1.1 × 10^6^ (8.0 × 10^5^)	5.8	122	1037	8.5	5.92 × 10^7^
		Acetate	No addition	No addition	9.5	1.6 × 10^6^ (2.4 × 10^5^)	6.3	124	1046	8.4	5.44 × 10^7^
		Formate	No addition	No addition	9.5	3.7 × 10^5^ (2.6 × 10^6^)	4.2	124	400.2	3.2	2.21 × 10^7^
	CSW1.1	^–^	^–^	^–^	12.4	1.68 × 10^5^		830	1232	1.5	5.02 × 10^5^
		CO_2_	No addition	No addition	7	2.5 × 10^5^ (1.6 × 10^5^)	0.5	126	135.1	1.1	2.00 × 10^5^
		CH_4_	No addition	No addition	12.5	1.8 × 10^6^ (2.4 × 10^5^)	3.4	124	140.6	1.1	1.06 × 10^6^
		Acetate	No addition	No addition	12.5	1.7 × 10^6^ (6.7 × 10^5^)	3.3	124	103.9	0.8	4.90 × 10^5^
		Formate	No addition	No addition	12.5	1.4 × 10^6^ (4.3 × 10^5^)	3.0	123	120	1.0	1.01 × 10^5^

*H_2_ was used as electron donor in all treatments*.

a *number in parenthesis = standard deviation*.

b *n, numbers of generations*.

– *, original field sample, no additions*.

For CSWold, addition of nutrients had a positive effect on growth in comparison with the amendments without nutrients. When N/P solution was added to the CO_2_ incubations, the end point cell counts increased and the numbers of generations were four times higher (Table 2). The DNA yield in these incubations increased from 0.2 to 1.0 ng/mL of sample and 16S rRNA gene copies increased an order of magnitude. The effect of nutrients in the acetate treatments for CSWold was not as clear, while the numbers of cells per mL of samples were an order of magnitude higher (*p*-value = 0.018) and the numbers of generations increased upon the addition of nutrients, the DNA yield decreased from 3.6 to 2.8 ng/mL of sample and the 16S rRNA gene copy numbers decreased an magnitude lower than in the treatment with nutrients (Table 2).

#### August microcosms

In the microcosms established with samples collected in August 2013, we investigated the effect of different carbon sources in growth (Table 2). Based on end point cell counts (Table 2), as well as on DNA yield and 16S rRNA gene copies per mL of sample (Table 2), acetate and methane favored growth in all treatments, followed by formate.

Even though no additional electron acceptor or nutrients were added to these incubations, growth was observed in most treatments, suggesting that the fluids from CROMO have enough electron acceptors and nutrients to support microbial growth. For both N08B and CSWold fluids, when comparing acetate treatments with additional electron acceptors (March treatments) with the treatments without (August treatments), the difference in end point cell counts is not statistically significant (*p-value > 0.09* for both samples).

#### Microcosm sequence analysis

At the end of the incubations, 16S rRNA gene sequences were obtained from the DNA extracted. Sequence analysis from the March microcosms (Table 3) indicate that the dominant sequence for both samples, CSWold and N08B were closely related to *D. alkaliphilus*, an alkaliphilic organism isolated from a soda lake in Mongolia (Sorokin et al., 2008). This organism can grow autotrophically with H_2_ as electron donor and thiosulfate and polysulfide as electron acceptors. Sequences related to this organism have been previously found in different continental sites (Brazelton et al., 2013; Suzuki et al., 2013; Tiago and Veríssimo, 2013) and are suggested to be one of the dominant organisms in serpentinizing environments (Suzuki et al., 2013). The sequences from CSWold and N08B were 97.3 and 98.9 similar, respectively, to an environmental sequence from another continental serpentinizing site in Portugal (Tiago and Veríssimo, 2013) (Table 4).

**Table 3:** **End point sequences from microcosm experiments and closest relative according to NCBI blast database^a^**.

**Month**	**Sample**	**Treatment**	**Fragment size (bp)**	**Closest relative**
March	CSWold	Acetate, H_2_, Na_2_S_2_O_3_/Na_2_S	1320	96% *Dethiobacter alkaliphilus* AHT1 (NR_044205)
March	NO8B	Acetate, H_2_, Na_2_S_2_O_3_/Na_2_S	1320	97% clone clone CVCloAm3Ph44 (AM778015)
August	N08B	CO_2_, H_2_	383	87% Uncultured bacterium (JX223906)
August	N08B	CH_4_, H_2_	1414	99% Comamonadaceae bacterium B1 DNA, complete genome (AP014569)
August	N08B	Acetate, H_2_	393	96% Comamonadaceae bacterium B1 DNA, complete genome (AP014569)
August	N08B	Formate, H_2_	394	87% *Brachymonas denitrificans* a337 (EU433324)
				86% Comamonadaceae bacterium B1 DNA, complete genome (AP014569)
August	CSWold	CO_2_, H_2_	382	95% *Dethiobacter* sp. Clone (FR695974)
August	CSWold	CH_4_, H_2_	343	91% Comamonadaceae bacterium B1 DNA, complete genome (AP014569)
August	CSWold	Acetate, H_2_	506	*Pseudomonas* sp. Clone (KC166766)
August	CSWold	Formate, H_2_	489	100% bacterial clone from Cambrian Sandstone subsurface (DDMA2E11)
August	CSW1.1	CO_2_, H_2_	1412	99% Comamonadaceae bacterium B1 DNA, complete genome (AP014569)
August	CSW1.1	CH_4_, H_2_	760	98% Comamonadaceae bacterium B1 DNA, complete genome (AP014569)
August	CSW1.1	Acetate, H_2_	432	99% Comamonadaceae bacterium B1 DNA, complete genome (AP014569)
August	CSW1.1	Formate, H_2_	674	99% Comamonadaceae bacterium B1 DNA, complete genome (AP014569)

a *NCBI database as of July 2014*.

*GenBank Accession numbers for these sequences are KM983097-KM983110*.

**Table 4:** **Percent identity between end point sequences from March microcosms (from Table 3), most abundant OTUs from tag sequence data, and reference sequences**.

	**CSWold**	**N08B**	***Dethiobacter alkaliphilus (NR044205)***	**CVCloAm3Ph44^a^ (AM778015.1)**
CSWold				
N08B	97.9			
*Dethiobacter alkaliphilus* (NR044205)	98.4	96.8		
CVCloAm3Ph44 (AM778015.1)	97.3	98.9	96.8	
OTU002	98.1	99.7	97.1	99.2

a *Clone from serpentinization-driven Cabeco de Vide Aquifer in Portugal (Tiago and Veríssimo, 2013)*.

Upon addition of different carbon sources, the end point sequence results showed a more diverse set of populations were enriched (Table 3). A newly isolated and described strain proposed as the new genus *Serpentinomonas*, related to Comamonadaceae bacterium B1, was the most abundant sequence in all three samples, (Suzuki et al., 2014). Two representative sequences, one from CSW1.1 and one from N08B microcosm, with CO_2_ and acetate respectively, shared 100% identity to Comamonadaceae bacterium B1 (Table 5). These sequences also share 100% identity with OTU001 (Table 5), which is the most abundant OTU in the 16S rRNA tag sequence libraries from our samples (Supplementary Figure 2) suggesting the abundance and importance of this organism in the CROMO fluids and its key role in the serpentinite- hosted microbiome.

**Table 5:** **Percent identity between two representative end point sequences from the August microcosms, most abundant OTUs from tag sequence data, and reference sequences**.

	**CSW1.1 CO_2_**	**N08B Acetate**	**Bacterium B1^a^ (AP014569)**	**Bacterium A1^b^ (AP014568)**	**CVCloAm1Ph9^c^ (AM777999)**
CSW1.1_CO_2_					
N08B_Acetate	100				
Bacterium_B1 (AP014569)	100	100			
Bacterium_A1 (AP014568)	99.7	99.7	99.7		
CVCloAm1Ph9 (AM777999)	100	100	100	99.7	
OTU001	100	100	100	99.7	100

a Comamonadaceae bacterium B1 and

b *Comamonadaceae bacterium A1 from continental serpentinizing site at The Cedars (Suzuki et al., 2014)*.

c *Clone from serpentinization-driven Cabeco de Vide Aquifer in Portugal (Tiago and Veríssimo, 2013)*.

## Discussion

Comprehensive geochemical analyses of well waters collected from CROMO in March and August 2013 revealed a high pH, methane-rich subsurface environment, relatively enriched in dissolved nutrients and DOC and with higher conductivity relative to previously studied continental serpentinites and in contrast to a control more neutral well. These data could reflect the degree to which the well waters interact with buried marine sedimentary organic matter, similar to previous observations at The Cedars (Morrill et al., 2013), or could reflect mixing with fluids percolating through serpentinite soils. Both of these scenarios are the topic of ongoing investigation. Nevertheless, these data provide a detailed basis to explore environmental controls upon microbial communities structure and to guide laboratory experiments.

Tag sequence data of 16S rRNA genes from the CROMO wells shows that high pH well waters harbor exceptionally low microbial diversity relative to more moderate systems, consistent with previous studies (Brazelton et al., 2013; Suzuki et al., 2013). The predominant taxa in tag sequence surveys were related to *Serpentinomonas*-like Betaproteobacteria, and *Dethiobacter*-like Clostridia, also consistent with earlier studies.

The microcosm results from the March 2013 experiments show that over the course of 3 months, nutrient amendments did not have a significant influence upon microbial growth or population structure. While this is in sharp contrast to suggestions from other continental serpentinite systems, these results are consistent with the relatively high concentrations of dissolved N and P species in the CROMO fluids. On the other hand, additions of thiosulfate and sulfide to the microcosms led to the almost exclusive enrichment of populations related to *D. alkaliphilus*. The microcosm set-up for the March experiments was similar to the media used by Sorokin et al. (2008) for the initial enrichment of the sulfate reducing *D. alkaliphilus*. While it is unsurprising that the amendments to serpentinite fluids led to the enrichment of this group, it is interesting that the microcosms enriched almost exclusively for this clade, whether or not acetate or nutrients were added. The enrichment of *D. alkaliphilus*-like organisms from low salinity serpentinite fluids also provides a fascinating contrast to explore physiological adaptations to high pH.

August microcosm experiments investigated microbial growth responses to a range of carbon sources commonly found in serpentinizing fluids. In general, results of these experiments show that both methane and acetate stimulated growth, consistent with the observation of these carbon species in the natural well water. While the stimulation of growth by acetate addition is consistent with our understanding of both *Serpentinomonas* and *Dethiobacter* metabolism, growth following the addition of methane was surprising relative to earlier studies. Previous studies of continental serpentinites have provided scant evidence for biological methane cycling using either taxonomic or function gene approaches (Blank et al., 2009; Brazelton et al., 2012; Tiago and Veríssimo, 2013). It is possible that metabolic processing of methane is occurring by cryptic microbial populations present at low level within the microcosm experiments, supplying localized resources for the abundant betaproteobacterial communities. Alternatively, the *Serpentinomonas*-like species may have metabolic versatility beyond that which is already recognized, and which has not yet been deduced from genomic studies. Direct measurements of methane concentrations and oxidation rates are needed in order to confirm the importance of this process in the system.

It is important to note that although autotrophic treatments were the less favored in terms of growth, the end point pH of these incubations dropped considerably, which might have exerted some additional stress to the microbial community. Additional experiments to determine the effect of pH in these high pH adapted microbial communities should be done in order to better understand carbon fixation and microbial response to stress in the system.

Data from the current work provides a basis to guide the enrichment of the predominant microbial populations found both at CROMO and elsewhere, so that detailed comparative and physiological studies can be conducted and so that details about adaptation and gene expression can be elucidated. The data also provide a basis to guide culture-independent studies of microbial community composition in serpentinite, and identify carbon and sulfur species as potential important components. Future work using isolates obtained from these approaches can be used to understand the subtleties of microbial physiological responses in these systems, and clarify important gene targets. The current study provides one of the first attempts to connect observational data with experimental data in serpentinite ecosystems.

### Conflict of interest statement

The authors declare that the research was conducted in the absence of any commercial or financial relationships that could be construed as a potential conflict of interest.

## Supplementary material

Supplementary Figure 1 **Diversity of 16S rRNA gene OTUs (97% sequence similarity) from fluids collected at CROMO in March 2013**. Alpha-diversity, as displayed by rarefaction **(A)**, Beta-diversity, displayed in a community-dissimilarity dendrogram calculated from the Morisita–Horn index **(B)**.

Supplementary Figure 2 **Diversity of OTUs (97% similarity) belonging to the classes Betaproteobacteria and Clostridia within CSW1.1, N08B, and CSWold**. The Betaproteobacteria in CSW1.1 and N08B are dominated by a single OTU (OTU001) belonging to the family Comamonadaceae. The same OTU makes up 45% of the Betaproteobacteria in CSWold.
