# Supplementary figures and images for: Insights into environmental controls on microbial communities in a continental serpentinite aquifer using a microcosm-based approach
Source: Front Microbiol. 2014 Nov 14;5:604. doi: 10.3389/fmicb.2014.00604 (PMC4231944; doi:10.3389/fmicb.2014.00604)

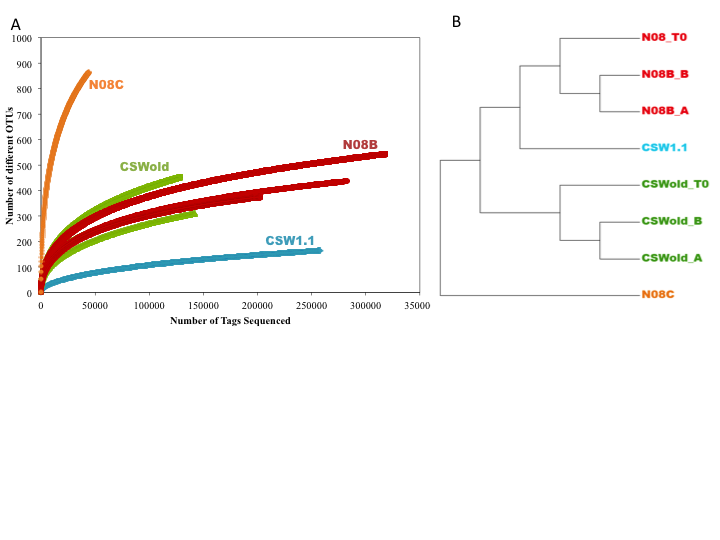

Supplement: Supplementary Figure 1 — Diversity of 16S rRNA gene OTUs (97% sequence similarity) from fluids collected at CROMO in March 2013. Alpha-diversity, as displayed by rarefaction (A), Beta-diversity, displayed in a community-dissimilarity dendrogram calculated from the Morisita–Horn index (B). [file Image1.TIF]

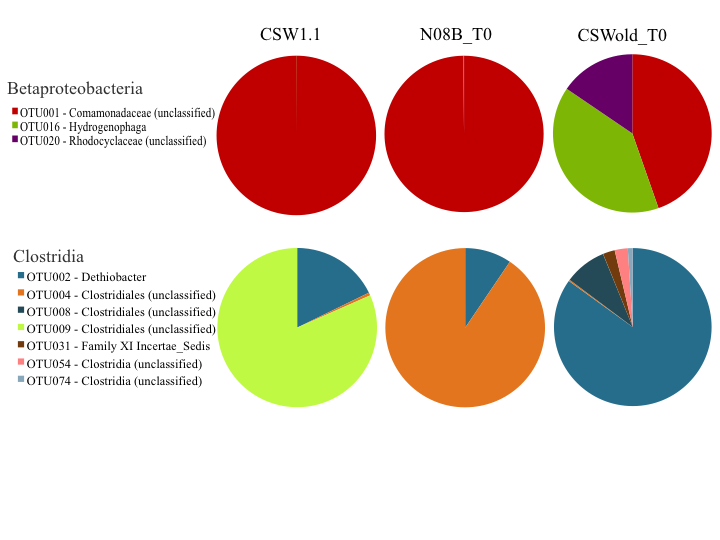

Supplement: Supplementary Figure 2 — Diversity of OTUs (97% similarity) belonging to the classes Betaproteobacteria and Clostridia within CSW1.1, N08B, and CSWold. The Betaproteobacteria in CSW1.1 and N08B are dominated by a single OTU (OTU001) belonging to the family Comamonadaceae. The same OTU makes up 45% of the Betaproteobacteria in CSWold. [file Image2.TIF]
